# Astragaloside IV alleviates heart failure by promoting angiogenesis through the JAK-STAT3 pathway

**DOI:** 10.1080/13880209.2019.1569697

**Published:** 2019-03-24

**Authors:** Yan-Bo Sui, Yu Wang, Li Liu, Feng Liu, Yi-Qing Zhang

**Affiliations:** a First Unit of Department of Cardiology, First Affiliated Hospital of Heilongjiang University of Chinese Medicine, Harbin, China;; b Heilongjiang University of Chinese Medicine, Harbin, China;; c Third Unit of Department of Cardiology, First Affiliated Hospital of Heilongjiang University of Chinese Medicine, Harbin, China;; d Fourth Unit of Department of Cardiology, First Affiliated Hospital of Heilongjiang University of Chinese Medicine, Harbin, China

**Keywords:** Left coronary artery ligation, CD31, VEGF, RNA interference

## Abstract

**Context:** Heart failure (HF) is one of the most serious diseases worldwide. Astragaloside IV (ASI) is widely used for the treatment of cardiovascular disease in China.

**Objective:** To evaluate the protective effect of ASI on the HF in a Sprague–Dawley rat model of left coronary artery ligation, and investigate the angiogenesis-related mechanisms.

**Materials and methods:** Left coronary artery was ligated to induce a rat model of HF, and the rats were treated with vehicle (saline) or different doses of ASI (0.1, 0.3 and 1 mg/kg/day) by oral gavage for 6 weeks. Cardiac function was evaluated by echocardiography. Infarct size was determined by triphenyltetrazolium chloride staining. Cardiac vascular density was analyzed by microangiography. Real-time PCR, Western blot and chromatin immunoprecipitation were performed to investigate the mechanisms.

**Results:** ASI treatment improved the body weight and survival rate of HF rats, as well as the cardiac function of HF rats, with significantly improved ejection fraction (75.27 ± 5.75% vs. 36.26 ± 4.14%) and fractional shortening (45.39 ± 3.66% vs. 17.88 ± 1.32%). ASI reduced the infarct size of the HF rats by 47%. ASI promoted angiogenesis, with increased vascular density (2.08-fold) and induced mRNA expression of CD31 (1.81-fold) and VEGF (2.70-fold) in the ischemic heart. Furthermore, ASI induced the phosphorylation of JAK (1.89-fold) and STAT3 (2.95-fold), as well as the activity of VEGF promoter which was regulated by STAT3.

**Discussion and conclusions:** ASI alleviated HF by promoting angiogenesis through JAK-STAT3 pathway, providing novel alternative strategies to prevent HF in the future.

## Introduction

Heart failure (HF), often referred to as congestive HF, occurs when the heart is unable to pump sufficiently to maintain blood flow to meet the body's needs (Jessup and Brozena 2003). It affects about 40 million people globally, causing it to be the most serious health issue in both developed and developing countries. Many factors such as ischemic heart disease, hypertension, diabetes, dyslipidemia and smoking can increase the risk for development of HF (Cohn et al. 1996; Bui et al. 2011).

Recent research found that angiogenesis-mediated recovery of the microvasculature is stimulated by pharmacological or genetic intervention that may have a greater potential in the therapy of HF (Ylä-herttuala and Alitalo 2003; van der Laan et al. 2009). Angiogenesis is the physiological process through which new blood vessels form from preexisting vessels (Carmeliet 2005). Vascular endothelial growth factor (VEGF) is a crucial regulator of angiogenesis, which is produced by cells and can stimulate the formation of blood vessels (Ferrara et al. 2003; Olsson et al. 2006). Signal transducer and activator of transcription 3 (STAT3) is an extensively investigated nuclear factor which is activated by cytokine receptors via Janus kinase (JAK) (Levy and Darnell 2002). STAT3 exerts crucial effects on angiogenesis in heart pathogenesis, and is a potential molecular target of angiogenesis-mediated therapy (Osugi et al. 2002; Hilfiker-kleiner et al. 2004).

Astragaloside IV (ASI) is the major active constituent of *Astragalus propinquus* Schischkin (Fabaceae), a widely used herb for the treatment of cardiovascular disease in China (Miller 1998; Sinclair 1998). Previous studies reported effects of ASI on HF in rats (Zhao et al. 2009; Wang et al. 2010; Dong et al. 2017). In addition, ASI stimulated angiogenesis in human umbilical vein endothelial cells, including cell proliferation, migration, and tube formation (Zhang et al. 2011; Wang et al. 2013). However, whether proangiogenic mechanism is involved in the effect of ASI on HF is still unknown. Here, we tried to clarify the mechanism of ASI on the HF protection in a rat model of left coronary artery ligation.

## Materials and methods

### Drugs

ASI was purchased from Sigma-Aldrich (St. Louis, MO, USA), and the purity is more than 98%. ASI was dissolved in 20% dimethylsulfoxide (DMSO) and diluted with saline for animal study. For *in vitro* assays, ASI was dissolved in DMSO, and the final DMSO concentration did not exceed 0.1% (v/v).

### Cell culture

Primary human umbilical vein endothelial cells (HUVEC) were purchased from ScienCell (Carlsbad, CA, USA) and cultured in endothelial cell culture medium (ScienCell) in a humidified 5% CO_2_ air atmosphere at 37 °C. Only cells from passages 3–6 were used for experiments.

### RNA interference

Primary HUVEC were seeded in six-well plates the day before transfection. RNAiMAX (Invitrogen, Carlsbad, CA) was used to transfect 50 nM of STAT3 siRNA (Santa Cruz Biotechnology, Santa Cruz, CA, USA) into the cells in OPTI-MEM Medium (Invitrogen) for 48 h. Control siRNA containing a scrambled sequence (Santa Cruz) was used as a negative control.

### Tube formation assay

The transfected primary HUVEC were plated into 48-well plates that were pre-coated with Matrigel (BD Biosciences, San Jose, CA, USA) and then treated with 20 μM of ASI for 6 h. Tubular structures were photographed and quantified by measuring the length of each tube using the ImageJ software (NIH, Boston, MA, USA). The tube length in three random fields from each well was calculated to determine the average length.

### Experimental animals

Adult male Sprague–Dawley (SD) rats (200–220 g) were purchased from Vital River (Beijing, China), and kept under standard conditions of animal room (temperature 26 °C; humidity 52–60%). All animal care and experimental protocols complied with the Animal Management Rules of the Ministry of Health of the People’s Republic of China and approved by the Institutional Animal Care and Use Committee of Heilongjiang University of Chinese Medicine.

### Induction of chronic HF model

After anesthetized with pentobarbital sodium (30 mg/kg), the SD rats were connected to an electrocardiograph (ECG) recorder and treated with tracheal intubation, then subjected to the surgery of left coronary artery ligation (*N* = 90) or sham (*N* = 10). The left coronary artery was ligated permanently to induce HF model as described previously (Fletcher et al. 1981; Pfeffer et al. 1985). In brief, after cutting off the third rib, the left descending anterior coronary was ligated 2–3 mm near its origin with a 6.0 silk thread. Successful ligation was verified by the colour change immediately in the ischemic area (anterior ventricular wall and the apex) and the occurrence of arrhythmias (ST-segment elevation). The chest was closed in layers and the skin was sterilized with povidone iodine. Twenty-four hours after the surgery, the survival rate was 67%. Sham-operated animals were performed by thoracotomy to open the pericardium only, with no ligation around the left anterior descending artery.

### Drug treatment

After 24 h, the survival rats were randomly divided into five groups: (1) sham group (*N* = 10); (2) model group (*N* = 15); (3) low dose of ASI (ASI L)-treated group (0.1 mg/kg/day) (*N* = 15); (4) middle dose of ASI (ASI M)-treated group (0.3 mg/kg/day) (*N* = 15); (5) high dose of ASI (ASI H)-treated group (1 mg/kg/day) (*N* = 15). Animals were treated with vehicle (saline) or ASI by oral gavage for 6 weeks. During the treatment, body weight of the rats was measured every two or three days, and the dosage of drug was adjusted according to the body weight. The death of animals was recorded every day.

### Echocardiography

Six weeks after the surgery, rats were anesthetized with pentobarbital sodium (30 mg/kg) and placed on a heating pad. Cardiac function of the rats was dynamically evaluated by echocardiography using Vevo770 (Visual Sonics Inc., Toronto, Canada) with a 716 probe. The transducers with frequency of 17.5-MHz for ventricular structure provided spatial resolutions. Left ventricular internal dimension in systole (LVIDs) and left ventricular internal dimension in diastole (LVIDd) were obtained from the M-mode tracings, while ejection fraction (EF) and fractional shortening (FS) were derived automatically by the High-Resolution Electrocardiograph system.

### Infarct size

After echocardiography measurements, six animals for each group were sacrificed and the hearts were excised immediately, and then stored at –80 °C for freeze after PBS washing. Each heart was cut manually into 6–8 transverse slices. After dipping in 1% triphenyltetrazolium chloride (TTC) solution at 37 °C for 30 min, these slices were flushed with saline and then fixed in 4% paraformaldehyde for 30 min. Next, the slices were placed on a glass slide and photographed by digital camera, then analyzed by ImageJ software.

### Microangiography

At the termination, 4 animals for each group were anesthetized by pentobarbital sodium (30 mg/kg), and catheters (PE-50; BD Biosciences) were implanted into the left common carotid artery at 2.5–3 cm to reach the mitral valve. Then, heparinized saline (10 U/mL), nitroglycerin (100 μg/mL), and barium sulphate (size, 1 μm; 0.1 g/mL) were manually injected successively to perfuse the vessels in the heart. The isolated hearts of the rats were placed in an X-ray chamber, and angiogram images were captured with an In Vivo FX PRO system (Carestream, Rochester, NY, USA). Vascular density was determined by pixel analysis using the software ImageJ.

### Real-time polymerase chain reaction (RT-PCR)

Trizol reagent (Takara, Dalian, China) was used for isolating total RNA from ventricular tissue. 50–100 mg of tissue was directly lysed by mixing with 1 mL of Trizol reagent and homogenized using a homogenizer. Then 0.2 mL of chloroform was added to the homogenized sample, and incubated for 20 min at room temperature. Subsequently, RNA was precipitated by mixing with isopropyl alcohol. Total RNA yield was quantified by microplate reader (Molecular Devices, Sunnyvale, CA, USA) measured at 260 nm. Then mRNA was isolated from total RNA by using Oligo (dT), and reverse transcribed into first-strand complement DNA (cDNA) and amplified using a PrimeScript 1st strand cDNA synthesis kit (Takara). Reaction system included 2 μL of cDNA, 12.5 μL of 2 × SYBR Green 1 Master Mix (Takara), and 1 μL of each primer. The PCR condition was as follows: pre-incubation at 95 °C for 30 s, followed by 40 cycles of denaturation at 95 °C for 5 sec, and annealing/extension at 60 °C for 30 s using iQ5 RT-PCR detection system (Bio-Rad, Hercules, CA, USA). The primers were listed as below:

CD31 Forward 5′-TATCCAAGGTCAGCAGCATCGTGG-3′, Reverse 5′-GGGTTGTCTTTGAATACCGCAG-3′

VEGF Forward 5′-CTGTGTGCCCCTGATGCGATGC-3′, Reverse 5′-CCTCCGGACCCAAAGTGCTCTG-3′

### Western blot

The fresh ventricular tissue was homogenized by a rotor-stator homogenizer in ice-cold lysis buffer (Beyotime, Shanghai, China). After boiling with loading buffer (Beyotime), denatured proteins were separated in SDS-PAGE gel, and transferred onto PVDF membrane. The membrane was blocked with nonfat milk, followed by incubation with primary antibody of p-JAK, JAK, p-STAT3, STAT3, CD31, GAPDH (Cell Signaling, Danvers, MA, USA) and VEGF (Santa Cruz) at 4 °C overnight. HRP-conjugated secondary antibody (Cell Signaling) was used to incubate the membrane for another 1 h the next day. Immobilon solution (Millipore, Billerica, MA, USA) was poured on the membrane to develop the band captured by FluorChem Image System (Alpha Innotech, Santa Clara, CA, USA).

### Chromatin immunoprecipitation (ChIP)

ChIP was performed with an Agarose ChIP Kit (Pierce) according to the manufacturer’s instructions. A ChIP-grade primary antibody against STAT3 was purchased from Cell Signaling. Immunoprecipitated DNA was purified with DNA Clean-Up Column (Tiangen, Beijing, China) and then quantitated by RT-PCR. The primer of VEGF promoter was listed below:

Forward 5′-CTGGCCTGCAGACATCAAAGTGAG-3′, Reverse 5′-CTTCCCGTTCTCAGCTCCACAAAC-3′

### Statistical analysis

All values were presented as means ± standard deviations (SD). One-way analysis of variance (ANOVA) was used to examine statistical comparisons among groups. Two-tailed Student’s *t-*test was used to examine statistical significance of differences between two groups. Kaplan–Meier survival curves were compared by use of a log-rank test. A difference with *p* value less than 0.05 was considered to be statistically significant.

## Results

### ASI improved the body weight and survival rate of HF rats

**Figure 1.**
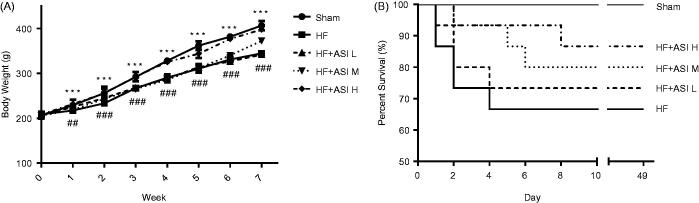
Body weight and survival rate of the rats. HF was induced by ligation of left coronary artery, and rats were treated with vehicle (saline) or different doses of ASI (0.1, 0.3 and 1 mg/kg/day) by oral gavage for 6 weeks. A. Body weight. B. Kaplan–Meier survival curve. Data are presented as Mean ± SD. *N* = 10–15. ##*p* < 0.01, ###*p* < 0.001 vs. sham group. ***p < 0.001 vs. model group.

The ligation of left coronary artery led to a significant reduction of body weight (Figure 1(A)). ASI increased the body weight of the HF rats with a dose-dependent manner. High dose of ASI could significantly increase the body weight since the first week post the surgery (*p* < 0.001 vs. model group). Furthermore, compared with the low survival rate of HF rats in model group (10/15), all three doses of ASI improved the survival rate of HF rats (11/15 for ASI L, 12/15 for ASI M, 13/15 for ASI H; Figure 1(B)). But the improvement of the survival rate is not significant (*p* > 0.05).

### ASI improved the cardiac function of HF rats

**Figure 2.**
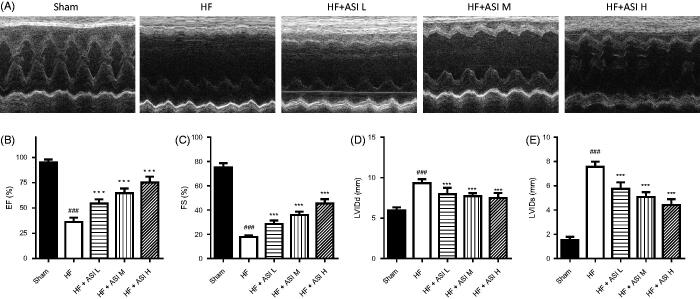
Cardiac function of the rats. After the treatment for 6 weeks, cardiac function of the rats was dynamically evaluated by echocardiography. A. Representative echocardiographic records obtained from a short-axis mid-ventrical view of the hearts. B–E. Statistical analysis of the data obtained or derived from original echocardiographic records. Data are presented as Mean ± SD. *N* = 6. ###*p* < 0.001 vs. sham group. ***p < 0.001 vs. model group.

Permanent ligation of left coronary artery led to the cardiac function injured, with much lower EF than that in sham group (36.26 ± 4.14% vs. 95.23 ± 2.80%; *p* < 0.001; Figure 2). ASI improved the cardiac function in a dose-dependent manner, with significantly increased EF (54.45 ± 4.08% for ASI L, 64.65 ± 4.66% for ASI M, 75.27 ± 5.75% for ASI H; *p* < 0.001 vs. model group). Similarly, ASI also significantly increased FS compared with that in model group (*p* < 0.001). In addition, impaired cardiac function led to enlarged heart chamber, with increased LVIDs and LVIDd (*p* < 0.001 vs. sham group). All three doses of ASI could significantly attenuate the increased LVID (*p* < 0.001 vs. model group).

### ASI reduced the infarct size of the heart

**Figure 3.**
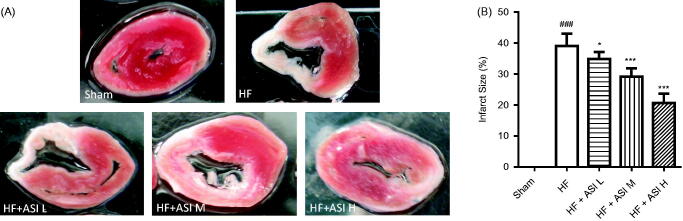
Infarct size of the hearts. At the termination, the hearts were excised, and infarct size was determined by TTC staining. A. Representative photograph of infarct size. B. Statistical analysis of infarct size. Data are presented as Mean ± SD. *N* = 6. ###*p* < 0.001 vs. sham group. *p < 0.05 vs. model group, ***p < 0.001 vs. model group.

Permanent ligation of left coronary artery caused the ischemia of left ventricular myocardium, followed by serious infarct (Figure 3(A)). ASI significantly reduced the infarct size in the left ventricle compared with that in model group (*p* < 0.001 vs. model group for ASI H and ASI M; *p* < 0.05 vs. model group for ASI L; Figure 3(B)).

### ASI promoted angiogenesis in ischemic heart

**Figure 4.**
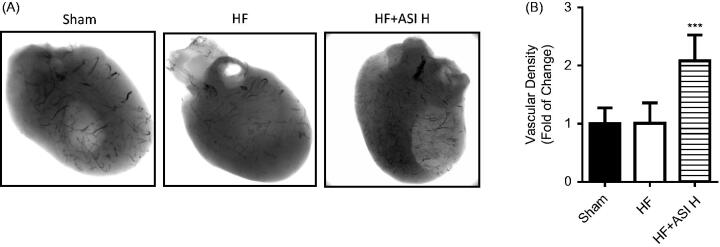
Vascular density of the hearts. At the termination, microangiography was performed to analyze the vascular density of the hearts. A. Representative photograph of vascular density. B. Statistical analysis of vascular density. Data are presented as Mean ± SD. *N* = 4. ***p < 0.001 vs. model group.

**Figure 5.**
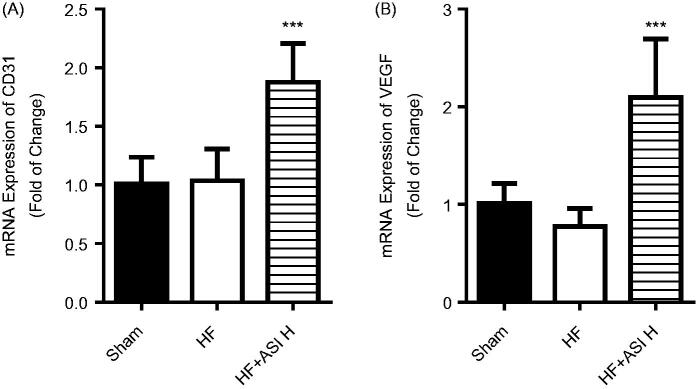
mRNA expression of CD31 and VEGF. The expression of mRNA in the ventricular tissue was determined by real-time PCR. A. mRNA expression of CD31. B. mRNA expression of VEGF. Data are presented as Mean ± SD. *N* = 6. ***p < 0.001 vs. model group.

Angiographic data showed increased vascular density of ischemic heart in ASI-treated rats compared with that in vehicle-treated rats (2.08 ± 0.44-fold change; *p* < 0.001; Figure 4). Next we analyzed the mRNA expression of CD31, a specific endothelial cell marker, and vascular endothelial growth factor (VEGF), and found that ASI also led to significantly increased mRNA expression of CD31 and VEGF (Figure 5).

### JAK-STAT3 pathway was involved in proangiogenic effects of ASI

**Figure 6.**
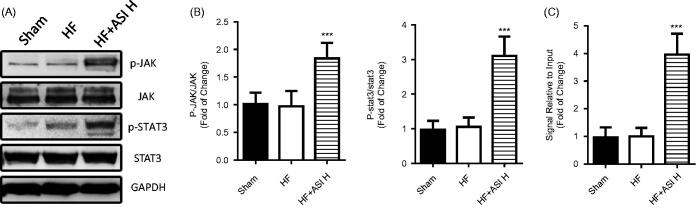
JAK-STAT3 pathway. The protein expression and phosphorylation of JAK and STAT3 in the ventricular tissue was determined by Western blot. A. Representative photographs of Western blot. B. Statistical analysis of Western blot. C. Activity of VEGF promoter. The nuclear extract was isolated from the ventricular tissue and used to perform chromatin immunoprecipitation with an STAT3 antibody. The change of downstream promoter was analyzed by real-time PCR. Data are presented as Mean ± SD. *N* = 6. ***p < 0.001 vs. model group.

Western blot showed significantly induced phosphorylation of JAK and STAT3 by ASI treatment (Figure 6(A,B)). Instead, ASI did not change the total expression of JAK and STAT3. We continued to perform ChIP using STAT3 antibody to observe a significant activation of VEGF promoter after ASI treatment (Figure 6(C)).

**Figure 7.**
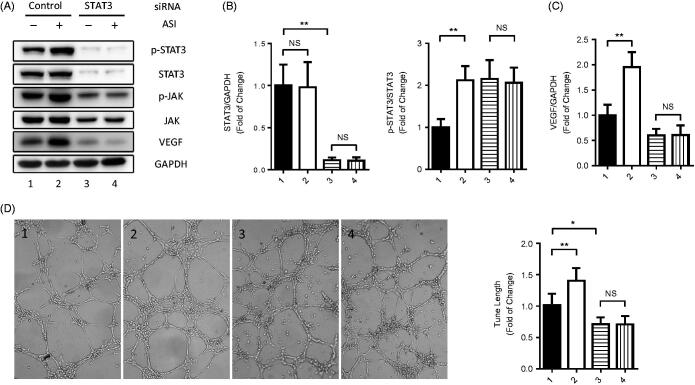
RNA interference in primary HUVEC. STAT3 silencing was performed by siRNA transfection using an RNAiMAX kit for 48 h before the primary HUVEC were lysed for Western blot (A–C) or seeded on the gelled Matrigel and treated with 20 μM of ASI for 6 h for tube formation assay (D–E). A. Representative photographs of Western blot. B. Statistical analysis of STAT3 and p-STAT3. C. Statistical analysis of VEGF. D. Representative photographs of tube formation assay. E. Statistical analysis of tube length. All experiments were repeated at least three times. Data are presented as Mean ± SD. *N* = 6. *p < 0.05, **p < 0.01. NS, no significance.

We transfected STAT3 siRNA into primary HUVEC to intervene STAT3 expression (Figure 7(A–C)), and observed that the tube formation of primary HUVEC in Matrigel were significantly inhibited by the interference (Figure 7(D,E)). In the control siRNA group, 20 μM of ASI promoted the tube formation of primary HUVEC, while in the STAT3 siRNA group, the proangiogenic effect of ASI were abolished because of the deficit of STAT3 expression (Figure 7(D,E)). Besides, the interference of STAT3 siRNA also abolish the effect of ASI on the expression of VEGF (Figure 7(C)), but had no influence on CD31 expression (data not shown).

## Discussion

A large number of clinical trials which were carried out in Chinese hospitals reported that *Astragalus membranaceus* could improve the left ventricular function and mitigate the cardiac hypertrophy in HF patients (Yuang 2003). Recently, increasing evidence showed that ASI, a saponin, is the active ingredient of *A. membranaceus* in the treatment of HF (Zhao et al. 2009; Wang et al. 2010; Dong et al. 2017). Previous studies reported that ASI improved EF, FS, and LV dimensions in rat and mouse model of HF which was induced by left coronary artery ligation (Zhao et al. 2009; Wang et al. 2010; Dong et al. 2017). In our study, ASI improved the body weight and survival rate of HF rats, as well as the cardiac function of HF rats, which was consistent with the previous data.

In previous research, fibroblast growth factor or VEGF was injected into the ischemic myocardium of patients with HF as protein or plasmid, which led to promoted angiogenesis, reduced symptoms, and improved myocardial perfusion (Chantal et al. 1998; Losordo et al. 1998). These results of angiogenesis-mediated therapy shed new light on HF treatment. Several *in vitro* studies reported that ASI stimulated angiogenesis in human umbilical vein endothelial cells, including cell proliferation, migration, and tube formation, but the authors did not investigate the proangiogenic mechanism of ASI in *in vivo* study (Zhang et al. 2011; Wang et al. 2013). In our study, we first reported ASI alleviated HF by promoting angiogenesis in a rat model, with increased vascular density and induced mRNA expression of CD31 and VEGF in the ischemic heart after ASI treatment. However, we did not observe significant difference in biomarker levels between sham and model group. Compared with the previous studies using an acute myocardial infarction model (Omura et al. 2001; Fan et al. 2015; Wang et al. 2017), our study induced a chronic heart failure model with left coronary artery ligation for 6 weeks, in which myocardial infarction underwent compensatory recovery.

STAT3 plays an important role in cell survival, and it is required for myocardial capillary growth after ischemic injury (Hilfiker-kleiner et al. 2004). STAT3 deletion in cardiomyocytes results in reduced capillarization of the left ventricle (Hilfiker-kleiner et al. 2004). In contrast, cardiac specific STAT3 activation promotes vascular formation in the heart (Osugi et al. 2002). Wang reported that ASI stimulated angiogenesis and increased nitric oxide accumulation by activating JAK/STAT3 and ERK pathway in human umbilical vein endothelial cells (Wang et al. 2013). Here, we also observed an induced phosphorylation of JAK and STAT3 in the ischemic ventricular tissue after ASI treatment. Furthermore, using ChIP analysis, we discovered ASI treatment activated VEGF promoter via STAT3, in which the activation of VEGF promoter may be directly responsible for ASI-induced angiogenesis. We also transfected STAT3 siRNA into primary HUVEC to silence STAT3 expression and finally proved that the proangiogenic effects of ASI could be abolished by STAT3 siRNA interference, demonstrating the critical role of STAT3 in the proangiogenic effects of ASI.

## Conclusions

We first reported that ASI could alleviate HF by promoting angiogenesis in a rat model of left coronary artery ligation. Furthermore, we also clarified a critical role of JAK-STAT3 pathway in the proangiogenic effects of ASI.

## References

[CIT0001] Bui AL, Horwich TB, Fonarow GC. 2011. Epidemiology and risk profile of heart failure. Nat Rev Cardiol. 8:30–41. 21060326 10.1038/nrcardio.2010.165 PMC3033496

[CIT0002] Carmeliet P. 2005. Angiogenesis in life, disease and medicine. Nature. 438:932–936. 16355210 10.1038/nature04478

[CIT0003] Chantal L, Wickwar VB, Wlodek K. 1998. Induction of neoangiogenesis in ischemic myocardium by human growth factors: first clinical results of a new treatment of coronary heart disease. Circulation. 97:645–650. 9495299 10.1161/01.cir.97.7.645

[CIT0004] Cohn JN, Cohn JN, Cohn JN. 1996. Drug therapy – The management of chronic heart failure. New Engl J Med. 335:490–498. 8672155 10.1056/NEJM199608153350707

[CIT0005] Dong Z, Zhao P, Xu M, Zhang C, Guo W, Chen H, Tian J, Wei H, Lu R, Cao T. 2017. Astragaloside IV alleviates heart failure via activating PPARα to switch glycolysis to fatty acid β-oxidation. Sci Rep. 7:2691–269115. 28578382 10.1038/s41598-017-02360-5 PMC5457407

[CIT0006] Fan D, Takawale A, Shen M, Wang W, Wang X, Basu R, Oudit G, Kassiri Z. 2015. Cardiomyocyte a disintegrin and metalloproteinase 17 (ADAM17) is essential in post-myocardial infarction repair by regulating angiogenesis. Circ Heart Fail. 8:970–979. 26136458 10.1161/CIRCHEARTFAILURE.114.002029

[CIT0007] Ferrara N, Gerber HP, Lecouter J. 2003. The biology of VEGF and its receptors. Nat Med. 9:669–676. 12778165 10.1038/nm0603-669

[CIT0008] Fletcher PJ, Pfeffer JM, Pfeffer MA, Braunwald E. 1981. Left ventricular diastolic pressure-volume relations in rats with healed myocardial infarction. Effects on systolic function. Circ Res. 49:618–626. 7261261 10.1161/01.res.49.3.618

[CIT0009] Hilfiker-Kleiner D, Hilfiker A, Fuchs M, Kaminski K, Schaefer A, Schieffer B, Hillmer A, Schmiedl A, Ding Z, Podewski E, et al. 2004. Signal transducer and activator of transcription 3 is required for myocardial capillary growth, control of interstitial matrix deposition, and heart protection from ischemic injury. Circ Res. 95:187–195. 15192020 10.1161/01.RES.0000134921.50377.61

[CIT0010] Jessup M, Brozena S. 2003. Heart failure. New Engl J Med. 348:2007–2018. 12748317 10.1056/NEJMra021498

[CIT0011] Levy DE, Darnell JE. 2002. Stats: transcriptional control and biological impact. Nat Rev Mol Cell Biol. 3:651. 12209125 10.1038/nrm909

[CIT0012] Losordo DW, Vale PR, Symes JF, Dunnington CH, Esakof DD, Maysky M, Ashare AB, Lathi K, Isner JM. 1998. Gene therapy for myocardial angiogenesis: initial clinical results with direct myocardial injection of phVEGF165 as sole therapy for myocardial ischemia. Circulation. 98:2800–2804. 9860779 10.1161/01.cir.98.25.2800

[CIT0013] Miller AL. 1998. Botanical influences on cardiovascular disease. Altern Med Rev. 3:422–431. 9855567

[CIT0014] Olsson AK, Dimberg A, Kreuger J, Claesson-Welsh L. 2006. VEGF receptor signalling - in control of vascular function. Nat Rev Mol Cell Biol. 7:359–371. 16633338 10.1038/nrm1911

[CIT0015] Omura T, Yoshiyama M, Ishikura F, Kobayashi H, Takeuchi K, Beppu S, Yoshikawa J. 2001. Myocardial ischemia activates the JAK-STAT pathway through angiotensin II signaling in in vivo myocardium of rats. J Mol Cell Cardiol. 33:307–316. 11162135 10.1006/jmcc.2000.1303

[CIT0016] Osugi T, Oshima Y, Fujio Y, Funamoto M, Yamashita A, Negoro S, Kunisada K, Izumi M, Nakaoka Y, Hirota H, et al. 2002. Cardiac-specific activation of signal transducer and activator of transcription 3 promotes vascular formation in the heart. J Biol Chem. 277:6676. 11744720 10.1074/jbc.M108246200

[CIT0017] Pfeffer MA, Pfeffer JM, Steinberg C, Finn P. 1985. Survival after an experimental myocardial infarction: beneficial effects of long-term therapy with captopril. Circulation. 72:406–412. 3891136 10.1161/01.cir.72.2.406

[CIT0018] Sinclair S. 1998. Chinese herbs: a clinical review of Astragalus, Ligusticum, and Schizandrae. Altern Med Rev. 3:338–344. 9802911

[CIT0019] van der Laan AM, Piek JJ, van Royen N. 2009. Targeting angiogenesis to restore the microcirculation after reperfused MI. Nat Rev Cardiol. 6:515–523. 19528962 10.1038/nrcardio.2009.103

[CIT0020] Wang C, Li Y, Yang X, Bi S, Zhang Y, Han D, Zhang D. 2017. Tetramethylpyrazine and astragaloside IV synergistically ameliorate left ventricular remodeling and preserve cardiac function in a rat myocardial infarction model. J Cardiovasc Pharmacol. 69:34–40. 27676326 10.1097/FJC.0000000000000437

[CIT0021] Wang SG, Xu Y, Chen JD, Yang CH, Chen XH. 2013. Astragaloside IV stimulates angiogenesis and increases nitric oxide accumulation via JAK2/STAT3 and ERK1/2 pathway. Molecules (Basel, Switzerland). 18:12809–12819. 10.3390/molecules181012809 PMC6270590 24135938

[CIT0022] Wang WT, Zhao ZY, Han YM, Wei-Ren XU, Tang LD. 2010. Effects of Astragaloside IV derivative on heart failure in rats. Chin Herb Med. 2:48–53.

[CIT0023] Ylä-Herttuala S, Alitalo K. 2003. Gene transfer as a tool to induce therapeutic vascular growth. Nat Med. 9:694–701. 12778168 10.1038/nm0603-694

[CIT0024] Yuan GP. 2003. Evaluation of efficacy of astragalus injection in treatment of patients with chronic heart failure. Chin J Clin Pharmacol Ther. 8:710–711.

[CIT0025] Zhang L, Liu Q, Lu L, Zhao X, Gao X, Wang Y. 2011. Astragaloside IV stimulates angiogenesis and increases hypoxia-inducible factor-1α accumulation via phosphatidylinositol 3-kinase/Akt pathway. J Pharmacol Exp Ther. 338:485–491. 21576377 10.1124/jpet.111.180992

[CIT0026] Zhao Z, Wang W, Wang F, Zhao K, Han Y, Xu W, Tang L. 2009. Effects of Astragaloside IV on heart failure in rats. Chin Med. 4:6. 19338675 10.1186/1749-8546-4-6 PMC2674594

